# m^6^A‐mediated regulation of crop development and stress responses

**DOI:** 10.1111/pbi.13792

**Published:** 2022-02-28

**Authors:** Leilei Zhou, Guangtong Gao, Renkun Tang, Weihao Wang, Yuying Wang, Shiping Tian, Guozheng Qin

**Affiliations:** ^1^ 74519 Key Laboratory of Plant Resources Institute of Botany Chinese Academy of Sciences Haidian District, Beijing China; ^2^ University of Chinese Academy of Sciences Beijing China

**Keywords:** N^6^‐methyladenosine, m^6^A machineries, crop development, stress responses, crop improvement

## Abstract

Dynamic chemical modifications in eukaryotic messenger RNAs (mRNAs) constitute an essential layer of gene regulation, among which N^6^‐methyladenosine (m^6^A) was unveiled to be the most abundant. m^6^A functionally modulates important biological processes in various mammals and plants through the regulation of mRNA metabolism, mainly mRNA degradation and translation efficiency. Physiological functions of m^6^A methylation are diversified and affected by intricate sequence contexts and m^6^A machineries. A number of studies have dissected the functional roles and the underlying mechanisms of m^6^A modifications in regulating plant development and stress responses. Recently, it was demonstrated that the human FTO‐mediated plant m^6^A removal caused dramatic yield increases in rice and potato, indicating that modulation of m^6^A methylation could be an efficient strategy for crop improvement. In this review, we summarize the current progress concerning the m^6^A‐mediated regulation of crop development and stress responses, and provide an outlook on the potential application of m^6^A epitranscriptome in the future improvement of crops.

## Introduction

Posttranscriptional or cotranscriptional RNA chemical modifications play essential roles in determining mRNA fates. Among the 160 distinct mRNA modifications identified so far, N^6^‐methyladenosine (m^6^A) is the most abundant and best‐characterized (Shao *et al*., 2021). m^6^A methylation is reversible *in vivo*, and functions under the synergistic effect of m^6^A methyltransferases (writers), demethylases (erasers) and reader proteins (readers), which install, remove and recognize m^6^A marks, respectively (Shi *et al*., 2019a). In mammals, m^6^A installation is mainly implemented by a methyltransferase complex, in which the METTL3/METTL14 (methyltransferase‐like 3/14) heterodimer constitutes the core component (Liu *et al*., 2014; Wang *et al*., 2016a, 2016b). METTL3 harbours the methyl‐transfer activity and functions as a catalytic subunit, while METTL14 facilitates the binding of the complex to the targeted transcripts (Wang *et al*., 2016a, 2016b). In addition, other subunits including Wilm’s tumour 1‐associating protein (WTAP) (Ping *et al*., 2014), Vir like m^6^A methyltransferase associated (VIRMA) (Yue *et al*., 2018), Zinc finger CCCH domain‐containing protein 13 (ZC3H13) (Wen *et al*., 2018) and RNA‐binding motif protein 15 (RBM15) (Patil *et al*., 2016) were also revealed to be functionally important for the m^6^A deposition. As for m^6^A removal, the fat mass and obesity‐associated protein (FTO) and the alkylated DNA repair protein AlkB homolog 5 (ALKBH5), both containing the AlkB domain, represent the well‐studied demethylases in mammals (Jia *et al*., 2011; Zheng *et al*., 2013). m^6^A methyltransferases and demethylases act cooperatively to bring the reversibility for m^6^A methylation, accompanied by diversified deposition and distribution along transcripts (Shi *et al*., 2019a). To exert the physiological functions, m^6^A needs to be recognized by the reader proteins. Mammalian YTH‐domain proteins, including YTHDF1/2/3 (Dominissini *et al*., 2012; Wang *et al*., 2014, 2015) and YTHDC1/2 (Hsu *et al*., 2017; Xiao *et al*., 2016), as well as some specific ribonucleoproteins (Alarcón *et al*., 2015; Liu *et al*., 2015) or RNA binding proteins (RBPs) (Edupuganti *et al*., 2017; Huang *et al*., 2018; Wu *et al*., 2019), have the ability to recognize m^6^A sites, and further modulate the m^6^A‐modified transcripts by interacting with other functional regulatory proteins, thus were identified as the m^6^A reader proteins (Shi *et al*., 2019a). Currently, m^6^A has been unveiled to functionally modulate mRNA metabolism including mRNA stability (Huang *et al*., 2018; Wang *et al*., 2014), translation efficiency (Wang *et al*., 2015), alternative splicing (Zhao *et al*., 2014), and nuclear‐cytoplasm transport (Roundtree *et al*., 2017), thereby regulating multiple biological processes, such as embryonic and post‐embryonic development (Batista *et al*., 2014), cell circadian rhythms (Fustin *et al*., 2013), and cancer stem cell proliferation (Zhang *et al*., 2016). Given its prevalence and function diversity, m^6^A has also been extensively investigated, as an important epigenetic modification, in plants including Arabidopsis and various crops with important agronomic traits (Liang *et al*., 2020; Shao *et al*., 2021; Yue *et al*., 2019).

In the model plant *Arabidopsis thaliana*, the m^6^A machineries have been adequately identified and proved to harbour multiple physiological roles (Shao *et al*., 2021). The m^6^A methyltransferase complex regulates a variety of essential growth and development processes including embryo development (Zhong *et al*., 2008), root vascular formation (Růžička *et al*., 2017), seedling growth (Růžička *et al*., 2017) and apical dominance formation (Bodi *et al*., 2012). Importantly, the core subunit of m^6^A methyltransferase complex, FKBP12 interacting protein 37 KD (FIP37), which is a homolog of the mammalian WTAP, participates in maintaining the normal proliferation of shoot meristems by negatively regulating the mRNA stability of several key shoot meristem genes (Shen *et al*., 2016). The m^6^A demethylase AtALKBH10B‐mediated m^6^A removal elevates the mRNA stability of flower‐promoting genes, thereby positively regulating floral transition (Duan *et al*., 2017). These studies reveal that Arabidopsis m^6^A modification has a capacity to decrease mRNA stability, thereby reducing the mRNA abundance of specific genes. However, the YTH‐domain protein evolutionarily conserved C‐terminal region 2 (ECT2) was shown to stabilize m^6^A‐modified transcripts, and further modulate the development of trichome morphogenesis (Arribas‐Hernández *et al*., 2018; Scutenaire *et al*., 2018; Wei *et al*., 2018). Another YTH‐domain protein CPSF30‐L (the longer isoform of cleavage and polyadenylation specificity factor 30) is involved in regulating the alternative polyadenylation (Hou *et al*., 2021; Pontier *et al*., 2019; Song *et al*., 2021). Therefore, Arabidopsis m^6^A machineries could adopt diverse molecular mechanisms to cope with divergent physiological processes. Besides the development regulation, m^6^A modification also mediates the biotic and abiotic stress responses in Arabidopsis, partially by affecting the mRNA stability and alternative polyadenylation of targeted transcripts (Shao *et al*., 2021). The complexity of m^6^A functions and mechanisms implies that more pervasive investigations are needed to better understand its biological role in plants. The current mechanistic studies based on Arabidopsis provide an advantageous reference for other plant species including crops.

More recently, the human FTO‐mediated plant m^6^A demethylation caused a ~50% increase in field yield and biomass of rice and potato (Yu *et al*., 2021). These findings are spectacular and suggest that modulation of m^6^A methylation may hold potential in serving as a strategy to dramatically improve crop growth and yield. In this review, we summarize the current progress in understanding the m^6^A characteristics, the m^6^A‐mediated regulation of mRNA metabolism, and the mechanistic links of m^6^A with developmental processes and stress responses in crops. We also provide an outlook on potential applications and remaining challenges concerning m^6^A epitranscriptome in the future crop improvement.

## m^6^A characteristics in crops

### m^6^A distribution along transcripts in crops

**Figure 1:**
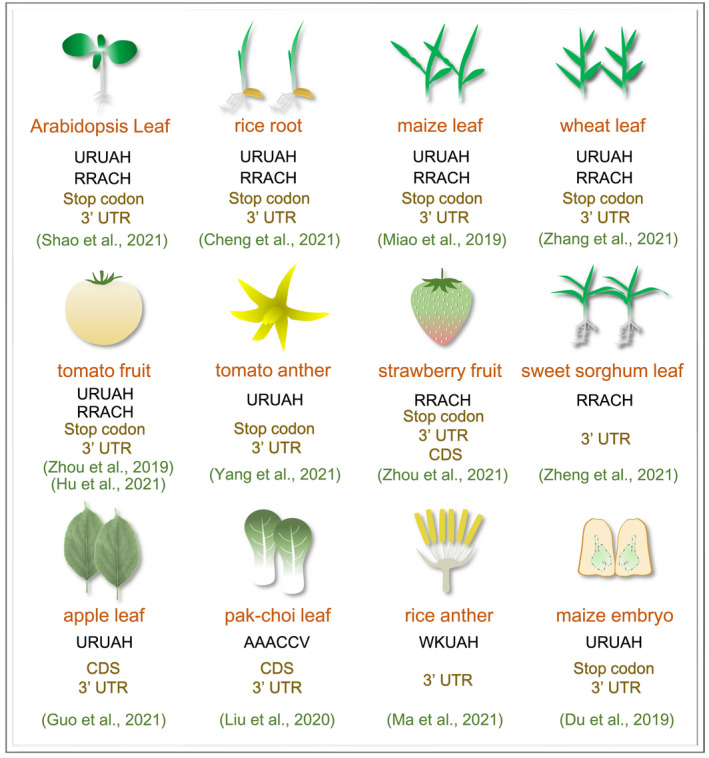
m^6^A motifs and distribution preferences along transcripts in various tissues of Arabidopsis and crops. R represents adenosine (A) or guanosine (G); H represents A, cytidine (C), or uridine (U); W represents A or U; K represents G or U; V represents A, G, or U. CDS, coding sequence. UTR, untranslated region. m^6^A motifs were predicted from m^6^A‐seq datasets that were performed with at least two independent biological replicates with standard m^6^A‐seq procedures.

With the application of high‐throughput m^6^A sequencing technology (m^6^A‐seq) (Dominissini *et al*., 2012; Meyer *et al*., 2012), the transcript‐specific m^6^A localization and enrichment have been uncovered at the transcriptome‐wide level in various plant species. In Arabidopsis, thousands of transcripts contain m^6^A modifications, which distribute preferentially around the stop codon or in the 3′ untranslated region (UTR) (Luo *et al*., 2014; Wan *et al*., 2015). This distribution preference is conserved among several important crops, including rice (*Oryza sativa*) (Cheng *et al*., 2021; Zhang *et al*., 2019a), maize (*Zea mays*) (Miao *et al*., 2020), wheat (*Triticum aestivum*) (Zhang *et al*., 2021b), tomato (*Solanum lycopersicum*) (Hu *et al*., 2021b; Yang *et al*., 2021; Zhou *et al*., 2019), and sweet sorghum (*Sorghum bicolor*) (Zheng *et al*., 2021) (Figure 1). A recent study that compared the m^6^A methylomes for 13 representative plant species spanning over half a billion years of evolution confirmed the conserved distribution of m^6^A modifications in the stop codon and 3′ UTR regions (Miao *et al*., 2022). Specially, the m^6^A modification in strawberry (*Fvesca vesca*) could also be highly enriched in the coding sequence (CDS) region adjacent to the start codon, besides the occurrence in the stop codon and 3′ UTR region (Zhou *et al*., 2021) (Figure 1). This unique CDS preference appears in the ripe strawberry fruit, but not the unripe fruit, indicating it is a ripening‐specific m^6^A characteristic (Zhou *et al*., 2021). Moreover, m^6^A modifications in apple (*Malus domestica*) and pak‐choi (*Brassica rapa*) leaves are most abundant in the CDS region, followed by the 3′ UTR region (Guo *et al*., 2021; Liu *et al*., 2020) (Figure 1). Therefore, m^6^A distribution around the stop codon or in the 3′ UTR could be conserved among various plants including Arabidopsis, rice, maize, wheat, tomato, sweet sorghum, strawberry, apple, and pak‐choi, while m^6^A deposition in the CDS region might be development stage‐specific or tissue‐specific. The inducements related to this distribution characteristic currently remain elusive. A recent study in Arabidopsis suggests that H3K36me2 histone marks contribute to the preferential m^6^A deposition in the 3’ UTR (Shim *et al*., 2020). This finding provides us valuable considerations in investigating the relevant mechanisms of m^6^A distribution in crops.

### m^6^A motifs in crops

The initial m^6^A‐seq analysis revealed that Arabidopsis m^6^A methylation occurs in a sequence context as RRACH (R represents adenosine (A) or guanosine (G); H represents A, cytidine (C), or uridine (U)) (Duan *et al*., 2017; Luo *et al*., 2014), the conserved m^6^A motif among mammals (Dominissini *et al*., 2012; Meyer *et al*., 2012). However, a subsequent study identified a plant‐specific m^6^A motif URUAH in Arabidopsis, which mainly locates within 3′ UTR and is targeted by the reader protein ECT2 (Wei *et al*., 2018). These results suggest that Arabidopsis possesses two different m^6^A motifs. Notably, a recent study claimed that URUAY is not an m^6^A motif, but it is rather enriched in the periphery of the canonical RRACH motifs (Arribas‐Hernández et al., 2021a). m^6^A marks in rice (Zhang *et al*., 2019a), maize (Miao *et al*., 2020), wheat (Zhang *et al*., 2021b), or tomato (Hu *et al*., 2021b; Yang *et al*., 2021; Zhou *et al*., 2019) fall into both the RRACH and URUAH motifs, as the model plant Arabidopsis (Figure 1). Strawberry (Zhou *et al*., 2021) and sweet sorghum (Zheng *et al*., 2021) harbour the conserved RRACH motif, while apple (Guo *et al*., 2021) was demonstrated to possess the plant‐specific URUAH motif (Figure 1). It is possible that the two distinct motifs may extensively exist in most of the crops, and could be individually identified in specific biological processes. Moreover, the consensus sequence of m^6^A in pak‐choi appears to be AAACCV (V means U, A, or G) (Liu *et al*., 2020), and four new m^6^A motifs were identified in rice anther, in which the WKUAH (W represents U or A; K means G or U) is the most abundant (Ma *et al*., 2021) (Figure 1). These findings suggest that m^6^A modifications in crops involve complicated sequence preferences. However, we currently do not know how m^6^A machineries achieve selectivity toward certain motif sequences to accomplish m^6^A installation, removal and recognition. One likely scenario is that they may be localized to diverse sequence contexts through interaction with RBPs that recognize distinct features of the transcripts.

## Regulation of m^6^A on mRNA metabolism in crops

m^6^A possesses multiple regulatory effects on mRNA metabolism in Arabidopsis, such as mRNA stability (Anderson *et al*., 2018; Arribas‐Hernández *et al*., 2021b; Duan *et al*., 2017; Kramer *et al*., 2020; Shen *et al*., 2016; Wei *et al*., 2018), transcriptome integrity (Pontier *et al*., 2019), and alternative polyadenylation (Hou *et al*., 2021; Hu *et al*., 2021a; Parker *et al*., 2020; Song *et al*., 2021). Currently, substantial progresses have been made in deciphering the influence of m^6^A methylation on crop mRNA metabolism, mainly mRNA degradation and translation (Shao *et al*., 2021).

### Modulation of mRNA stability in crops

Through the combination of transcriptome‐wide m^6^A‐seq and RNA‐seq analyses, the potential relationship between m^6^A modification and mRNA abundance has been revealed in rice (Cheng *et al*., 2021), maize (Du *et al*., 2020; Luo *et al*., 2020), tomato (Zhou *et al*., 2019), strawberry (Zhou *et al*., 2021), and pak‐choi (Liu *et al*., 2020) under divergent physiological circumstances. In the rice root threatened with cadmium stress (Cheng *et al*., 2021) or pak‐choi seedling treated with hot temperature (Liu *et al*., 2020), no exact correlation between m^6^A modification and mRNA abundance was determined, although thousands of transcripts exhibited differential m^6^A enrichment or gene expression under these stress conditions. However, a positive correlation between m^6^A methylation and mRNA levels was discovered in maize embryos cultured with 2,4‐D, an auxin analogue (Du *et al*., 2020). Moreover, m^6^A modification locating within the stop codon or 3’ UTR regions was shown to negatively regulate the mRNA abundance in normally growing maize seedling (Luo *et al*., 2020), tomato fruit (Zhou *et al*., 2019), and strawberry fruit (Zhou *et al*., 2021), while m^6^A enriching in the CDS region in ripe strawberry fruit tends to positively regulate the mRNA levels (Zhou *et al*., 2021). The different influences of m^6^A modification on mRNA abundance are correlated with the distinct effects of m^6^A on mRNA stability (Guo *et al*., 2021; Zhou *et al*., 2019, 2021). m^6^A deposition around the stop codon or within the 3′ UTR region possesses the ability to decrease mRNA stability, while m^6^A in the CDS region promotes mRNA stability. However, the underlying mechanisms need to be elucidated.

### Modulation of translation efficiency in crops

Although it is currently unclear whether m^6^A modification participates in mediating mRNA translation efficiency in Arabidopsis, several studies in crops obtained affirmative answers on this issue. In maize seedling, transcriptome‐wide polysome profiling analysis revealed that m^6^A possesses different effects on translation efficiency, depending on m^6^A abundances and positions in transcripts (Luo *et al*., 2020). Concretely, m^6^A modification nearby the start codon or in the 3′ UTR with low strength (low m^6^A enrichment value) tends to enhance mRNA translation, while m^6^A with excessive deposition (excessive m^6^A enrichment value) in the 3′ UTR decreases the translation efficiency (Luo *et al*., 2020). In strawberry fruit and apple leaf, m^6^A methylation was also demonstrated to facilitate mRNA translation (Guo *et al*., 2021; Zhou *et al*., 2021). More recently, the rice m^6^A adenosine methylase 2 (OsMTA2) was revealed to interact with the eukaryotic translation initiation factor 3 subunit h (OsEIF3h) (Huang *et al*., 2021), implying that m^6^A modification may modulate mRNA translation via the interactions between m^6^A machineries and translation factors, adopting the analogous molecular mechanism as the mammals (Lin *et al*., 2016; Wang *et al*., 2015).

## Regulatory mechanisms of m^6^A on crop developmental processes

Arabidopsis m^6^A marks have been demonstrated to modulate multiple physiological processes, reflecting its functional diversity in controlling plant development (Liang *et al*., 2020; Shao *et al*., 2021). Disruption of m^6^A machineries including m^6^A methyltransferase, demethylases and reader proteins could be an effective pointcut to explore the functional significance of m^6^A in both Arabidopsis and crops. Current researches have uncovered the m^6^A‐mediated regulation of key genes with critical roles in crop growth and development.

### Regulation of development in grain crops

**Figure 2:**
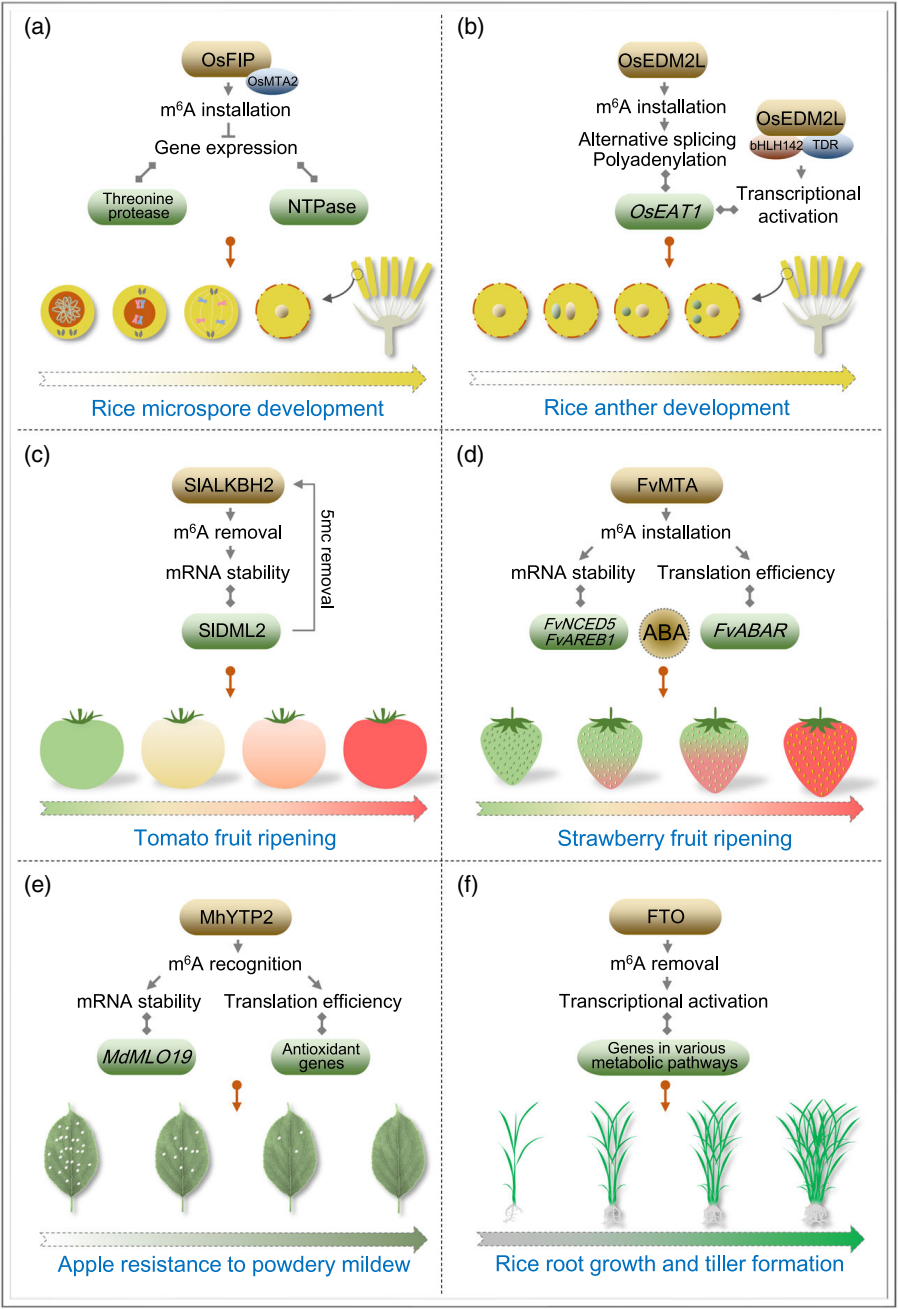
Function of m^6^A modification on crop growth and development or stress resistance. (a) Regulation of rice m^6^A methyltransferase subunit OsFIP on microspore development. OsFIP‐mediated m^6^A installation decreases the expression of genes encoding the threonine proteases and NTPases, thereby maintaining the normal development of rice microspores. *Os*, *Oryza sativa*. (b) Regulation of rice m^6^A methyltransferase‐like domain‐containing protein OsEDM2L on anther development. OsEDM2L‐mediated m^6^A installation facilitates the proper alternative splicing and polyadenylation of the *OsEAT1* mRNA that encodes a positive regulator of tapetal programmed cell death during the anther development. Moreover, OsEDM2L could directly activate the *OsEAT1* transcription by interacting with the transcription factors bHLH142 and TDR. (c) Regulation of tomato m^6^A demethylase SlALKBH2 on fruit ripening. SlALKBH2‐mediated m^6^A removal promotes mRNA stability of DNA demethylase gene *SlDML2*, a key ripening‐promoting gene, thereby facilitating fruit ripening. SlDML2 in turn acts on *SlALKBH2* to activate its transcription by DNA demethylation, representing an interplay between m^6^A RNA methylation and DNA methylation during tomato fruit ripening. *Sl*, *Solanum lycopersicum*. (d) Regulation of strawberry m^6^A methyltransferase FvMTA on fruit ripening. FvMTA‐mediated m^6^A installation promotes mRNA stability or translation efficiency of key genes in ABA pathway including *FvNCED5*, *FvAREB1* and *FvABAR*, thereby facilitating fruit ripening. *Fv*, *Fvesca vesca*. (e) Regulation of apple m^6^A reader MhYTP2 on leaf resistance to powdery mildew. MhYTP2‐mediated m^6^A recognition elevates the mRNA stability of *MdMLO19*, a positive regulator in powdery mildew resistance, and the translation efficiency of antioxidant genes, thereby enhancing the resistance of apple leaves to powdery mildew. *Mh*, *Malus hupehensis*; *Md*, *Malus domestica*. (f) Regulation of human m^6^A demethylase FTO on rice root growth and tiller formation. Heterologous expression of human FTO in rice promotes root growth and tiller formation by regulating the expression of genes in various metabolic pathways, therefore facilitating rice yield and biomass.

OsFIP, a homolog of the mammalian WTAP, was identified as one of the components in the m^6^A methyltransferase complex in rice (Zhang *et al*., 2019a). Functional investigation revealed that OsFIP mediates m^6^A deposition on transcripts of sporogenesis‐related genes encoding the NTPases and threonine proteases, leading to the acceleration of degradation of these transcripts to control microsporogenesis (Zhang *et al*., 2019a) (Figure 2a). Deficiency of the rice m^6^A methyltransferase‐like domain‐containing protein enhances downy mildew 2‐like (OsEDM2L) impaired tapetal programmed cell death (PCD) and causes defective pollen development, indicating its indispensable role for normal anther development (Ma *et al*., 2021). OsEDM2L could not only interact with the transcription factors basic helix‐loop‐helix 142 (bHLH142) and tapetum degeneration retardation (TDR) to activate the expression of eternal tapetum 1 (EAT1), a positive regulator of tapetal PCD, but also facilitate the suitable alternative splicing and polyadenylation of *OsEAT1* transcript by m^6^A installation, thus regulating the tapetal PCD and anther development (Ma *et al*., 2021) (Figure 2b). Moreover, the interaction of OsMTA2 with the OseIF3h suggests that OsMTA2 may participate in regulating OseIF3h‐mediated seedling growth and pollen development in rice, although the molecular mechanism is poorly understood (Huang *et al*., 2021).

In maize, m^6^A‐mediated post‐transcriptional regulation contributes to the heterosis in hybrid seedlings and the induction of callus cultured with the addition of auxin analogue 2,4‐D (Du *et al*., 2020; Luo *et al*., 2021). The former was predicted to correlate with the increased translation efficiency of m^6^A‐modified transcripts (Luo *et al*., 2021), while the latter may be caused by the elevated mRNA abundance of several key genes involved in the callus induction (Du *et al*., 2020).

### Regulation of development in horticultural crops

Fruit expansion represents an important process for fruit growth and development. It has been revealed that the overall m^6^A level increases during tomato fruit expansion, accompanied by the elevated m^6^A enrichments and transcript levels in several essential expansion‐related genes (Hu *et al*., 2021b). Exogenous treatment by 3‐deazaneplanocin A (m^6^A writer inhibitor) or meclofenamic acid (m^6^A eraser inhibitor) suppresses the expansion of fruit (Hu *et al*., 2021b), suggesting that m^6^A methylation participates in modulating the growth and development of tomato fruit. However, the underlying molecular mechanisms need to be clarified.

Recently, m^6^A methylation was reported to regulate fruit ripening, an important biological process for quality formation (Zhou *et al*., 2019, 2021). In the climacteric fruit tomato, m^6^A demethylase SlALKBH2 targets the DNA demethylase gene *DEMETER‐like DNA demethylase 2* (*SlDML2*), a key ripening‐promoting gene, and positively modulates its expression by elevating mRNA stability, thus accelerating tomato fruit ripening. Interestingly, SlDML2 can in turn act on *SlALKBH2* to activate its transcription by the repressive effect on DNA methylation (5‐methylcytosine, 5mC). These results suggest that SlALKBH2 and SlDML2 function synergistically during tomato fruit ripening, representing an internal correlation between m^6^A and 5mC (Zhou *et al*., 2019) (Figure 2c). In the non‐climacteric fruit strawberry, the m^6^A methyltransferase FvMTA was demonstrated to positively regulate fruit ripening (Zhou *et al*., 2021). FvMTA‐mediated m^6^A modification increases mRNA stability or translation efficiency of key genes in the ABA pathway including *9‐cis‐epoxycarotenoid dioxygenase 5* (*FvNCED5*), *ABA‐responsive element‐binding protein 1* (*FvAREB1*) and *putative ABA receptor* (*FvABAR*), which are essential for the ripening of strawberry fruit (Figure 2d). Strawberry genome contains four *SlDML2* homologs, among which two contain differential m^6^A peaks at the onset of fruit ripening. However, none of these genes exhibits a significant increase in mRNA abundance upon ripening initiation. In addition, no differential m^6^A modification was observed in the transcripts of DNA methyltransferase genes in the RNA‐directed DNA methylation (RdDM) pathway, which governs the reprogramming of DNA methylation during the ripening of strawberry fruit. This suggests that DNA methylation is dispensable for the FvMTA‐mediated fruit ripening in strawberry (Zhou *et al*., 2021). Thus, regulation of fruit ripening via m^6^A modification involves complicated molecular mechanisms and could be distinct among various fruits.

## Regulatory mechanisms of m^6^A on crop stress responses

Mutation of m^6^A writers in Arabidopsis induces significant changes in the expression of genes responsive to abiotic and biotic stresses, as revealed by transcriptome analysis (Anderson *et al*., 2018; Bodi *et al*., 2012; Hu *et al*., 2021a). Accordingly, the m^6^A‐mediated stress responses have been extensively studied in various crops, including tobacco (*Nicotiana tabacum*) (Li *et al*., 2018), rice (Cheng *et al*., 2021; Shi *et al*., 2019b; Tian *et al*., 2021; Zhang *et al*., 2021a), wheat (Sun *et al*., 2020; Zhang *et al*., 2021b), sweet sorghum (Zheng *et al*., 2021), tomato (Yang *et al*., 2021), apple (Guo *et al*., 2021; Mao *et al*., 2021), and pak‐choi (Liu *et al*., 2020).

### Regulation of the biotic stresses in crops

Tobacco mosaic virus (TMV) infection causes the increased expression of the potential demethylase genes, concomitant with the decreased expression of the potential methyltransferases, implicating the involvement of m^6^A in modulating the virus‐induced stress responses in tobacco (Li *et al*., 2018). In rice, the infection of rice stripe virus or rice black‐stripe dwarf virus (RBSDV) causes a dramatical increase in overall m^6^A methylation level, accompanied by the changed transcription in genes encoding m^6^A machineries, implying that m^6^A modification might be involved in the defence response against virus infection (Zhang *et al*., 2021a). In addition, when the m^6^A methyltransferase genes *LsMETTL3* and *LsMETTL14* from small brown planthopper (SBPH), the insect vector of RBSDV, were silenced, the titre of RBSDV in the midgut cells of SBPHs increased significantly, suggesting a negative correlation between the overall m^6^A levels and the virus replication (Tian *et al*., 2021). In watermelon, the infection by cucumber green mottle mosaic virus (CGMMV) leads to the decrease in global m^6^A level and the increase in expression of m^6^A demethylase gene *ClALKBH4B* (He *et al*., 2021). Some m^6^A‐modified transcripts related to plant immunity display altered m^6^A enrichments and transcript levels in response to CGMMV infection. It is probably that *ClALKBH4B* modulates the expression of the defence genes via m^6^A methylation, thereby participating in the regulation of watermelon against CGMMV infection (He *et al*., 2021).

The *Malus* YTH domain‐containing RNA binding protein 2 (MhYTP2) was demonstrated to positively regulate the apple resistance to powdery mildew (PM) caused by *Podosphaera leucotricha* (*P. leucotricha*) (Guo *et al*., 2021). MhYTP2 could decrease the mRNA stability of Mildew Locus O 19 (MdMLO19), a negative regulator of PM resistance, and improve the translation efficiency of antioxidant genes, thereby repressing the infection of *P*. *leucotricha* (Guo *et al*., 2021) (Figure 2e). The m^6^A methylation was also predicted to modulate fungus infection in rice, because virulence tests showed that the m^6^A machineries of *Pyricularia oryzae*, the causal agent of rice blast disease, were involved in virulence on rice (Shi *et al*., 2019b). In addition, wheat m^6^A methylation profiles revealed that thousands of transcripts, including those in plant defence responses and plant‐pathogen interaction pathway, display changed m^6^A abundances under the infection of wheat yellow mosaic virus, indicating that m^6^A marks would also participate in mediating wheat resistance to plant pathogens (Zhang *et al*., 2021b). These findings emphasize that the regulation of m^6^A in virus or fungus‐related biotic stress seems common in various crop species, although the underlying mechanisms need further investigation.

### Regulation of the abiotic stresses in crops

In addition to the role in biotic stress, m^6^A also modulates abiotic stress responses in crops. In rice, cadmium treatment induces the differential m^6^A modifications in thousands of transcripts in the root, suggesting that m^6^A may be associated with the abnormal root development caused by cadmium stress (Cheng *et al*., 2021). In wheat, genes of the m^6^A reader protein TaYTHs exhibit obvious expression changes in response to abiotic stresses including water and drought stresses (Sun *et al*., 2020). Salt stress induces a drastic alteration in m^6^A methylome in sweet sorghum, leading to the increase in m^6^A modification and mRNA stability of genes related to the salt resistance, which in turn positively regulated the tolerance to salt stress (Zheng *et al*., 2021). Furthermore, significant changes in m^6^A methylome profile, as well as its correlation with mRNA abundance, have been deciphered in pak‐choi seedling under heat stress (Liu *et al*., 2020), tomato anther under cold stress (Yang *et al*., 2021), and apple leaf under drought stress (Mao *et al*., 2021). These results revealed that m^6^A modification also participates in controlling the responses of crops to temperature and humidity‐induced stresses.

## Concluding remarks and future outlook

Rapid advances have been made in recent years in understanding the functional diversity of m^6^A marks, especially in some important biological processes. Physiological effects of m^6^A on plant development or stress resistance throughout the life cycle facilitate plant adaptation to the complicated and volatile ecological environment. In spite of this, one extremely essential issue is how to employ m^6^A investigations to increase the yield and quality of crops, known as crop improvement, thereby facilitating human health. Heterologous expression of human m^6^A demethylase FTO in rice and potato dramatically elevates the yield and biomass through the transcriptional modulation of genes involved in various metabolic pathways (Yu *et al*., 2021) (Figure 2f). Recent studies revealed that m^6^A methyltransferase or demethylase participates in modulating tomato and strawberry fruit ripening, an important process for fruit quality formation (Zhou *et al*., 2019, 2021). Moreover, m^6^A reader proteins were demonstrated to mediate the stress responses in both wheat and apple (Guo *et al*., 2021; Sun *et al*., 2020; Zhang *et al*., 2021b). These findings highlight the importance of m^6^A machineries in physiological regulations, and provide us the possibility for improving crop yield, quality, and stress resistance by controlling the bioactivity of m^6^A machineries.

Although m^6^A modification possesses great potential for crop improvement, there exist several major challenges. Firstly, modulation of the key components in m^6^A system may cause changes in the overall levels of m^6^A methylation, leading to unpredictable effects. Therefore, a transcriptome‐wide m^6^A modification map at single‐base resolution is necessary for precise m^6^A editing at specific sites without changing the overall levels of m^6^A or the primary sequence of genes with critical roles in crop development or stress response (Zheng *et al*., 2020). However, the current m^6^A modification maps constructed for plants can only display m^6^A modification in a range of 100–200 nucleotides. Current advances in high‐throughput m^6^A detection techniques at single‐base resolution, including miCLIP (m^6^A individual‐nucleotide‐resolution crosslinking and immunoprecipitation) (Linder *et al*., 2015), MAZTER‐seq (RNA digestion via m^6^A sensitive RNase and sequencing) (Garcia‐Campos *et al*., 2019), m^6^A‐REF‐seq (m^6^A‐sensitive RNA‐endoribonuclease‐facilitated sequencing) (Zhang *et al*., 2019b), and nanopore DRS (direct RNA sequencing) (Parker *et al*., 2020; Pratanwanich *et al*., 2021), may facilitate the precise identification of m^6^A sites. The second challenge is how to add or remove specific m^6^A modification sites in gene transcripts. The normal genome editing technique can only result in the addition or deletion of the gene sequences, but not the m^6^A modification sites. This could be resolved by the application of m^6^A editing, which makes it possible to precisely reconstruct the m^6^A marks at specific sites. As a new tool, m^6^A editing appears to be a fusion of m^6^A enzymes (writers or erasers) and clustered regularly interspaced short palindromic repeat (CRISPR)/CRISPR‐associated nuclease 9 (Cas9) technology. The m^6^A editing can add or remove m^6^A at specific sites under the guidance of single‐guide RNA and protospacer adjacent motif (Cox *et al*., 2017; Xu *et al*., 2019; Zheng *et al*., 2020). Recently, it is proposed that Cas13 has higher RNA target specificity and efficiency than Cas9, and is more suitable for the m^6^A editing system (Zheng *et al*., 2020). Moreover, CRISPR‐based prime editing harbours the ability to achieve the substitution, deletion or insertion of all single nucleotides in plant genomes, thus holding great potential in future m^6^A editing (Jin *et al*., 2021). The third challenge is that a number of m^6^A machineries, including writers, erasers, and readers, remain uncertain in crops. Although some m^6^A enzymes have been identified in several crops, such as rice, apple, and tomato, their numbers are few relative to those identified in animals. Besides the main challenges, many important questions require further explorations for better understanding and application of m^6^A in crop improvement. For example, what are the molecular mechanisms underlying the crosstalk among m^6^A machineries in the regulation of crop development and stress responses? Whether m^6^A participates in regulating the trade‐off between growth and resistance? In summary, the m^6^A‐mediated regulation in crops is an essential and complicated research area with nondeterminacy and many challenges.

In recent years, a series of bioinformatics tools were developed to simplify the analysis of m^6^A epitranscriptomics. RNAModR is the first publicly analytical toolkit suitable for m^6^A analysis (Evers *et al*., 2016). However, this tool focuses on the annotation of m^6^A distribution along transcripts, but lacks functions for m^6^A site calling. Subsequently, several prediction tools based on mammalian or yeast sequences were developed to predict m^6^A sites (Zhai *et al*., 2018). On that basis, a plant‐specific epitranscriptome package, named Plants Epitranscriptome Analysis (PEA), was exploited (Zhai *et al*., 2018). This bioinformatic toolkit is versatile and could perform the calling, prediction, and functional annotation of m^6^A sites produced from both common m^6^A‐seq and high‐throughput m^6^A detection techniques at single‐base resolution, such as miCLIP (Zhai *et al*., 2018). RNAmod is another versatile m^6^A toolkit, which facilitates the visualization and functional annotation of m^6^A modifications in multiple species including Arabidopsis (Liu and Gregory, 2019). However, whether PEA and RNAmod could be applied in numerous crop species needs to be determined. In addition, with the rapid development of m^6^A detection techniques, more convenient and integrated bioinformatic toolkits are required to be developed to achieve accurate identification of m^6^A sites, for example, those suitable for the third‐generation nanopore sequencing are currently vacant in plants.

## Conflict of interest

The authors declare that they have no competing interests.

## Authors’ contributions

GQ conceived the topic. LZ, GQ, GG, RT, WW, and YW wrote the manuscript. All authors read and approved the final manuscript.
